# Whole Genome Sequencing and Evolutionary Analysis of Human Respiratory Syncytial Virus A and B from Milwaukee, WI 1998-2010

**DOI:** 10.1371/journal.pone.0025468

**Published:** 2011-10-06

**Authors:** Cecilia Rebuffo-Scheer, Michael Bose, Jie He, Shamim Khaja, Michael Ulatowski, Eric T. Beck, Jiang Fan, Swati Kumar, Martha I. Nelson, Kelly J. Henrickson

**Affiliations:** 1 Midwest Respiratory Virus Program, Milwaukee, Wisconsin, United States of America; 2 Department of Pediatrics, Medical College of Wisconsin, Milwaukee, Wisconsin, United States of America; 3 Children's Research Institute, Milwaukee, Wisconsin, United States of America; 4 Fogarty International Center, National Institutes of Health, Bethesda, Maryland, United States of America; Centro Nacional de Microbiología - Instituto de Salud Carlos III, Spain

## Abstract

**Background:**

Respiratory Syncytial Virus (RSV) is the leading cause of lower respiratory-tract infections in infants and young children worldwide. Despite this, only six complete genome sequences of original strains have been previously published, the most recent of which dates back 35 and 26 years for RSV group A and group B respectively.

**Methodology/Principal Findings:**

We present a semi-automated sequencing method allowing for the sequencing of four RSV whole genomes simultaneously. We were able to sequence the complete coding sequences of 13 RSV A and 4 RSV B strains from Milwaukee collected from 1998–2010. Another 12 RSV A and 5 RSV B strains sequenced in this study cover the majority of the genome. All RSV A and RSV B sequences were analyzed by neighbor-joining, maximum parsimony and Bayesian phylogeny methods. Genetic diversity was high among RSV A viruses in Milwaukee including the circulation of multiple genotypes (GA1, GA2, GA5, GA7) with GA2 persisting throughout the 13 years of the study. However, RSV B genomes showed little variation with all belonging to the BA genotype. For RSV A, the same evolutionary patterns and clades were seen consistently across the whole genome including all intergenic, coding, and non-coding regions sequences.

**Conclusions/Significance:**

The sequencing strategy presented in this work allows for RSV A and B genomes to be sequenced simultaneously in two working days and with a low cost. We have significantly increased the amount of genomic data that is available for both RSV A and B, providing the basic molecular characteristics of RSV strains circulating in Milwaukee over the last 13 years. This information can be used for comparative analysis with strains circulating in other communities around the world which should also help with the development of new strategies for control of RSV, specifically vaccine development and improvement of RSV diagnostics.

## Introduction

Human Respiratory Syncytial Virus (RSV) is the leading cause of all lower respiratory-tract infections in infants and young children worldwide [1]. Population dynamics and poor socioeconomic conditions have contributed to the rise in the burden of RSV especially in developing countries where the population under 5 years of age has steeply increased within the last 30 years [2]. Hence, the establishment of new strategies for control of RSV has become an important task for public health authorities especially in the areas of prevention, diagnosis, and the development of an effective vaccine [3].

RSV is a member of the family *Paraxymoviridae,* subfamily *Pneumovirinae* that is differentiated into two groups (A and B) based on antigenic and genetic variability [4]–[6]. Studies of genetic variability among RSV strains belonging to each of these groups revealed the existence of distinct genetic patterns called genotypes [7]. To date, eight genotypes for RSV group A (named GA1 to GA7 and SAA1) [7]–[9] and eight for RSV group B (named BA, GB1 to GB4, SAB1 to SAB3) [7], [9], [10] have been described based on changes in the G gene. RSV B genotype BA was initially subdivided into six subgroups (BA1 to BA6) [11]–[13]. This number now increased to ten subgroups to include four recently emerged genotypes (BA7 to BA10) [14]. Two new variants of the RSV A genotype GA2 (NA1 and NA2) have only been described in Japan [15].

Most epidemiological and evolutionary studies reported worldwide for RSV have been based on the analysis of changes observed in just part of the G protein, which is one of the most variable among a total of 11 proteins encoded in the 15.2 Kb RSV genome [7], [9], [16], [17]. Those studies have provided a significant number of small fractions from different RSV genomes, however, only six complete genome sequences of original RSV strains have been published (GenBank: AY911262, M74568, U39662, FJ614813, AF013254 and AY353550). The most recent of which dates back 35 and 26 years for RSV A [18] and RSV B [19], respectively. The lack of efficient sequencing methods available for RSV has in part contributed to the limited knowledge of RSV evolution and virology at a genomic level.

This work was performed in an effort to develop a cost- and time-effective strategy for sequencing the whole genome of the RSV groups A and B facilitating a comprehensive study of the evolutionary characteristics of the RSV A and B strains circulating in Milwaukee over the last 13 years. Our work significantly increases the amount of complete genomic data that is available for both RSV groups A and B. Here we provide basic molecular characteristics of RSV that can be used for comparative analysis with strains circulating in other regions of the world, which should improve RSV control strategies, including vaccine development and improvement of RSV diagnostics.

## Materials and Methods

### Ethics Statement

All clinical samples were collected under protocols allowing sequencing approved by the Medical College of Wisconsin and Children's Hospital of Wisconsin institutional review boards. Some samples were approved to be collected retrospectively, de-identified, and did not require consent. The remaining samples were collected with written informed consent (from parent/guardian).

### Source of Clinical Samples and Isolation of the Virus

All specimens (Table 1) were collected from patients in the Milwaukee metro area by nasopharyngeal and nasal swabs and were immediately added to 3.0 ml of M4 viral transport medium (Remel, Lenexa, KS). Clinical samples were either collected with written informed consent (from parent/guardian) or received de-identified from the Children's Hospital of Wisconsin or Dynacare Laboratories with an IRB approved waiver of consent. Samples from 2006–2010 were determined to be positive by real-time RT-PCR [20], while samples from 1998 were determined to be positive by the Hexaplex assay [21].

**Table 1:** Milwaukee RSV strains sequenced.

RSV A			
Strain	Isolation Date	Source	% Genome Sequenced(1)
A/WI/629-3248/98	1/6/1998	Sample	99.4
A/WI/629-4071/98	1/9/1998	Isolate	99.6
A/WI/629-4111/98	1/27/1998	Sample	96.8
A/WI/629-3868/98	2/18/1998	Sample	95.0
A/WI/629-4110/98	3/5/1998	Sample	91.1
A/WI/629-3734/98	3/9/1998	Sample	98.4
A/WI/629-4239/98	3/21/1998	Sample	99.6
A/WI/629-4255/98	3/29/1998	Sample	95.5
A/WI/629-4266/98	4/14/1998	Sample	85.9
A/WI/629-4285/98	5/2/1998	Sample	92.7
A/WI/629-4302/98	5/17/1998	Sample	97.1
A/WI/629-4480/98	11/14/1998	Sample	59.9
A/WI/629-3/06-07	2006-7	Isolate	99.6
A/WI/629-9/06-07	2006-7	Sample	96.2
A/WI/629-17/06-07	2006-7	Isolate	99.6
A/WI/629-2/07	2007	Isolate	99.6
A/WI/629-9-2/07	2007	Isolate	99.6
A/WI/629-21/07	12/22/2007	Isolate	99.4
A/WI/629-22/07	12/28/2007	Isolate	99.6
A/WI/629-23/08	1/17/2008	Isolate	99.4
A/WI/629-DC9/08-09	2008-9	Isolate	99.6
A/WI/629-Q0154/10	1/13/2010	Sample	99.6
A/WI/629-Q0198/10	1/25/2010	Sample	98.2
A/WI/629-Q0282/10	2/16/2010	Sample	99.3
A/WI/629-Q0284/10	2/16/2010	Isolate	99.3

RSV B			
B/WI/629-5B/06-07	2006-7	Isolate	99.6
B/WI/629-12/06-07	2006-7	Sample	99.6
B/WI/629-15/06-07	2006-7	Sample	99.6
B/WI/629-DC1/08-09	2008-9	Sample	99.5
B/WI/629-DC2/08-09	2008-9	Sample	92.6
B/WI/629-DC8/08-09	2008-9	Sample	91.2
B/WI/629-DC10/08-09	2008-9	Sample	74.4
B/WI/629-Q0190/10	1/21/2010	Sample	99.6
B/WI/629-Q0306/10	3/3/2010	Sample	88.2

(1) The percent of the genome sequenced for each of the isolate/sample was calculated as the length (nt) of each isolate/sample sequenced divided by the total length (nt) of the prototype strain published for RSV. Previous to this calculation the sequences of the isolates/samples for RSV group A and group B were aligned to the prototype strains for RSV group A (M74568) and group B (AF013254) respectively.

In order to preserve the strains in this study we attempted to isolate RSV from many of the clinical samples by applying standard virus culture techniques using the epithelial cell line HEP-2 (ATCC, Manassas, VA). HEP-2 cells were inoculated with sample and incubated at 37°C with 5% CO2 in a humidified incubator for up to 10–14 days. Virus was harvested upon visualization of the characteristic RSV cytopathic effect, syncytium formation. Not all RSV strains could be isolated.

### Primer Design

PCR amplification primer pairs (IDT-DNA technologies, Coralville, IA) were designed for highly conserved regions to encompass both RSV A and RSV B strains based on sequence data from GenBank. Additional primers were designed from our sequence data as necessary. Degenerate bases were used in place of genetically variable bases across RSV A and RSV B strains when necessary. The RSV genome was divided into 12–13 overlapping segments for sequencing, with some segments utilizing several alternate primer pairs (Table S1 and Figure S1). Variability and length of a given segment dictated the number of internal overlapping sequencing primers designed for said segment to allow for complete coverage of the genome.

### RNA Extraction

RNA from clinical isolates and some clinical samples was extracted using the semi-automated NucliSENS easyMAG System (Biomerieux, Durham, NC) by combining 400 µL of sample with 1 mL of easyMAG Lysis Buffer for no less than 10 minutes. Samples were extracted following the manufacturer's generic automated 1.0 protocol for off-board lysis and nucleic acid was eluted into 50 µL of elution buffer. Extracted RNA from clinical samples and clinical isolates were handled identically post extraction and were used either directly or frozen at −80°C until needed.

Additional clinical samples were manually extracted with the RNeasy Mini Kit (QIAGEN, Valencia, CA). Samples were lysed by combining 400 µl of RLT buffer with 400 µL of clinical sample and incubating at ambient temperature for five minutes. Following the addition of 200 µL of 70% ethanol the lysate was processed according to the manufacturer's protocol. RNA was eluted in 50 µl RNase free water which was added to the spin column two minutes prior to centrifugation to increase elution efficiency.

### Two-step RT-PCR

Following extraction, 20 µL reverse transcription reactions were prepared containing 3 µL of RNA, 2.5 µM Random Hexamers, 4 mM dNTP Mix, 1x PCR Buffer II, 5 mM MgCl_2_, 2.5 U MuLV, and 1 U Rnase Inhibitor (Applied Biosystems, Carlsbad, CA). The reaction was performed on a GeneAmp 9700 thermocycler (Applied Biosystems) under the following conditions: 5 min at 22°C, 30 min at 42°C, and 5 min at 95°C. For every clinical sample or isolate, seven RT reactions were performed in order to prepare enough cDNA for 12–13 PCR reactions.

Each PCR reaction consisted of 10 µL of cDNA, a forward and reverse primer pair for one of the segments at 1 uM each, 1×PCR Buffer II, 1.25 mM MgCl_2_, (Applied Biosystems) 2.5 U FastStart Taq (Roche), with a total reaction volume of 50 µL. Samples were amplified on a GeneAmp 9700 thermocycler (Applied Biosystems) under the following conditions: 5 min at 95°C, six 3-step cycles at 95°C for 45 sec, 68°C (with a decrease of 2°C/cycle) for 5 min, and 72°C for 1 min, then five 3-step cycles of 95°C for 45 sec, 54°C for 2 min, and 72°C for 1 min, then 32 3-step cycles at 95°C for 45 sec, 52°C for 2 min, and 72°C for 1 min, and ended with a 10 min incubation at 72°C.

### Sequencing

The concentration of amplicon was determined using a DNA 7500 kit and DNA microfluidics chip as per the manufacturer's protocol on a 2100 Bioanalyzer (Agilent Technologies, Santa Clara, CA). The amplicon was then purified using the QIAquick PCR purification kit (Qiagen) following the manufacturer's protocol for a 50 µL sample.

Segments shown to contain multiple bands via the 2100 Bioanalyzer, were prone to sequence data with persistent high background. To rectify this issue, these samples underwent a gel purification step using a 1% agarose gel run with 1×TAE Buffer. The band of the appropriate size was excised and purified using Qiagen's QIAquick Gel Purification Kit following the manufacturer's protocol for PCR purification with spin columns. Sample purity and concentration was confirmed using the 2100 Bioanalyzer.

Purified amplicon was diluted based on the 2100 Bioanalyzer results to a final concentration of **∼**10 ng/rxn. Using a 384-well plate, 10 µL reactions were set up containing 5 µL of the diluted amplicon, a sequencing primer at 0.3 µM, 1×Big Dye buffer (Applied Biosystems), 0.225 µL of Big Dye Ready Reaction Mix v 3.1 (Applied Biosystems), and DEPC Water. When running all 12–13 segments for a complete RSV genome, 87–96 different primers were used (Table S1), allowing for the sequencing of 4 complete genomes for a given 384-well plate. The 384-well plate was sealed using a well-covering layer of Microseal ‘A’ Film (Bio-Rad, Hercules, CA) and an outer layer of Adhesive foil for Microplates (VWR, Radnor, PA). The sealed plate was pulse centrifuged and PCR was performed in a DNA Engine thermocycler (Bio-Rad) using the following protocol: 96°C for 5 sec then 40 3-step cycles at 96°C for 10 sec, 52°C for 5 sec, and 60°C for 4 min. After the sequencing reaction, the plate was pulse centrifuged and cleaned up on the automated Biomek FX fluid handler system (Beckman Coulter, Inc., Brea, CA) by first adding 20 µL of wash buffer (Millipore, Billerica, MA) to the samples. All plate contents were then moved to a 384-well filter plate and placed on an active vacuum manifold for 8 min. The filter plate was removed from the vacuum and 20 µL of injection solution (Millipore) was added to each well. The contents of the vacuum plate were transferred to a new 384-well plate and were pulse centrifuged. The sequencing reaction was read using a 3730 DNA Analyzer (Applied Biosystems). The sequencing was sometimes repeated with additional primers to close gaps remaining in the genomes.

### Sequence Data Analysis

Sequence assembly and consensus calling was performed using SeqMan Pro of the Lasergene 8 program suite (DNASTAR, Madison, WI). Reference sequences used for assembly were dependant on the subtype of RSV being analyzed. RSV A and B genomes were assembled using GenBank sequences U39662 and AY353550, respectively. Consensus sequences were exported for use in subsequent phylogenetic analyses.

### Phylogenetic Analysis

For each RSV subtype alignments for the whole genome and the coding sequence (CDS) of each protein (NS1, NS2, N, M, P, G, F, SH, M2-1, M2-2, and L) were made with additional published sequences available in GenBank using the multiple sequence alignment program MAFFT [22]. An additional alignment was made using the C-terminal region of the G protein coding sequences that has been described previously [7], [9], [16], [17]. Editing and reformatting of the alignments were made using CLC Sequence Viewer 6.4 (http://www.clcbio.com) and BioEdit 7.0.5.3 [23]. The best model of evolution for the sequencing data was determined by employing the GTR+G model of nucleotide substitution as determined by MODELTEST. This facilitated the inference of the evolutionary relationship for the complete data set analyzed in this work for RSV A and RSV B. Trees were made for each alignment using a Bayesian analysis with the program MrBayes (version 3.1.2) [24] with at least 1×10^6^ generations. For support, trees were also made using the neighbor-joining and maximum parsimony methods in PAUP* (portable version 4.0b10) [25]. Tree manipulations were made using the FigTree v1.3.1 program (available at http://tree.bio.ed.ac.uk/software/figtree/).

### Synonymous and Non-synonymous Mutations

Mutations in each CDS (CoDing Sequence) were analyzed by the method of Nei and Gojobori [26]. Codon aligned sequences for each genome were run through the SNAP program (http://www.hiv.lanl.gov/content/sequence/SNAP/SNAP.html) [27] to calculate the variability for each CDS in both RSV A and B. Alignments included the CDS sequences from the available whole genomes for RSV A (AY911262, FJ614813, M74568, and U39662) and RSV B (AF013254 and AY353550).

### Genotype Divergence

The intragenotype divergence was measured as the average of the synonymous versus non-synonymous mutation ratio (dS)/(dN) for the four RSV A genotypes found in Milwaukee between 1998–2010. The SNAP program [27] was used for these calculations.

### Intergenic Sequence Analysis

As a measurement of the variability of each intergenic (IG) region we calculated the percent identity of each IG region for each of the 33 stains compared to the correspondent IG sequences (IGS) of the prototype strain A2 (M74568) for RSV A and strain 9320/77 (AY353550) for RSV B. After alignment of the IGS of the prototype strain with the IGS of our strains, we counted the number of positions in our strains that are identical to the prototype sequence and divided by the total number of nucleotide positions in that IGS. This was examined for each one of the eight IGS connecting the following RSV genes (3′-NS1-NS2-N-P-M-SH-G-F-M2-5′). Since the M2 and L genes overlap there is no IGS between them to analyze.

### Gene Start and End Sequences

Using the whole genome alignments the gene start (GS) and gene end (GE) sequences were identified using previously identified sequences for the A2 strain for RSV A. Because not all GE sequences were described for RSV B we used either previously published sequences for strain 18537 or by comparison to the locations and sequences in RSV A.

### Non-coding Regions Analysis

As a measurement of conservation/variation of the 5′ and 3′ non-coding regions (NCR) of each RSV gene we calculated the percent of conserved nucleotides in each NCR for each of the 25 RSV A stains compared to the correspondent NCRs of the prototype strain A2 (M74568) for RSV A. After alignment of the NCRs of the prototype strain with the NCRs of our strains, we counted the number of positions in our strains that are identical to the prototype sequence divided by the total number of nucleotide positions in that NCR. The same conservation/variation of the 5′NCRs and 3′NCRs in the nine RSV B strains from Milwaukee was calculated but the strains were compared with each other instead of comparing with the RSV B prototype strain. This was examined for all eleven RSV genes excluding the GS, GE, start codon, and stop codon sequences.

### Entropy Plot and Graphical Representation of Variation and Conservation

Using BioEdit 7.0.5.3 [23] an entropy plot was calculated with the RSV A and B genome alignments to determine which positions had variation. The positional information was imported into Microsoft Excel. Corrected values were calculated for all positions that were missing information in any of the sequences. The corrected values, positions of coding regions, and positions of non-coding regions were plotted in a bar graph format.

Sequences obtained in the course of this study have been submitted to GenBank and assigned accession numbers: JF920046, JF920047, JF920048, JF920049, JF920050, JF920051, JF920052, JF920053, JF920054, JF920055, JF20056, JF920057, JF920058, JF920059, JF920060, JF920061, JF920062, JF920063, JF920064, JF920065, JF920066, JF920067, JF920068, JF920069, JF920070, JN032115, JN032116 JN032117 JN032118 JN032119, JN032120, JN032121, JN032122, JN032123.

## Results

### Phylogenetic Analysis

In this work a total of 25 RSV A and 9 RSV B strains were sequenced and analyzed. We were able to assemble a single contig covering the complete coding sequence for 13 of the 25 RSV A and for 4 of the 9 RSV B strains sequenced. The remaining 12 RSV A and 5 RSV B sequences cover the majority of the genome but have small gaps throughout the genome (Table 1).

**Figure 1:**
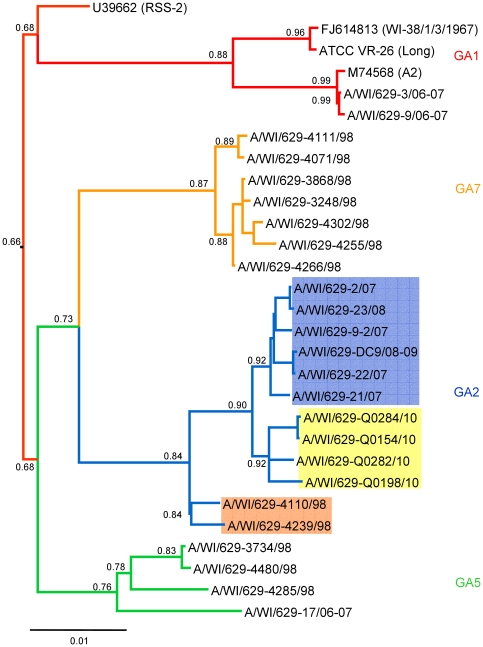
Phylogenetic tree of the RSV group A whole genome from Milwaukee and published strains. Branches are colored coded according to the four different genotypes: GA1 (red), GA2 (blue), GA7 (orange), GA5 (green). The GA2 sub-clades corresponding to the year of collection are highlighted (in the orange box are the strains collected in 1998, in the yellow box are the strains collected in 2010, and in the blue box are the strains collected between 2007–2009). The tree was calculated using 1×10^6^ generations and a burn in of 12 in MrBayes. Node values correspond to posterior probabilities.

**Figure 2:**
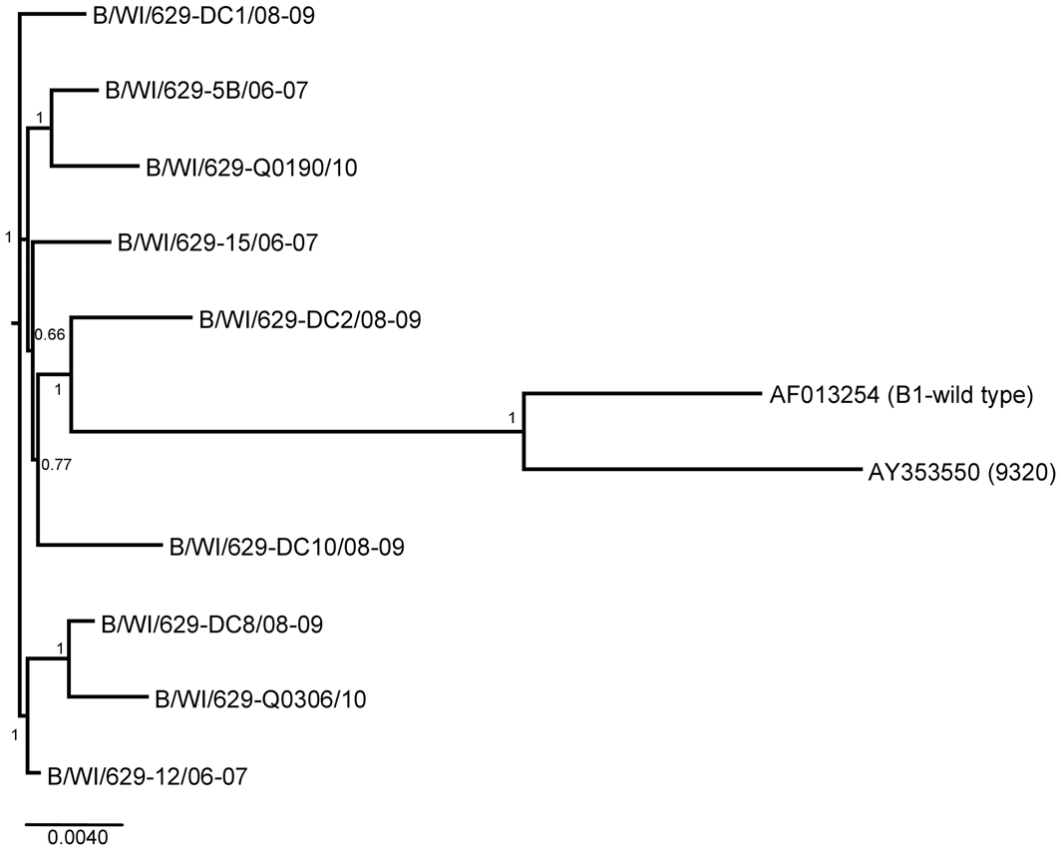
Phylogenetic tree of the RSV B whole genomes from Milwaukee and the two published genome sequences. The tree was calculated using 5×10^6^ generations in MrBayes and a burn in of 15. Node values are posterior probabilities. (In parenthesis are the published strain names).

**Figure 3:**
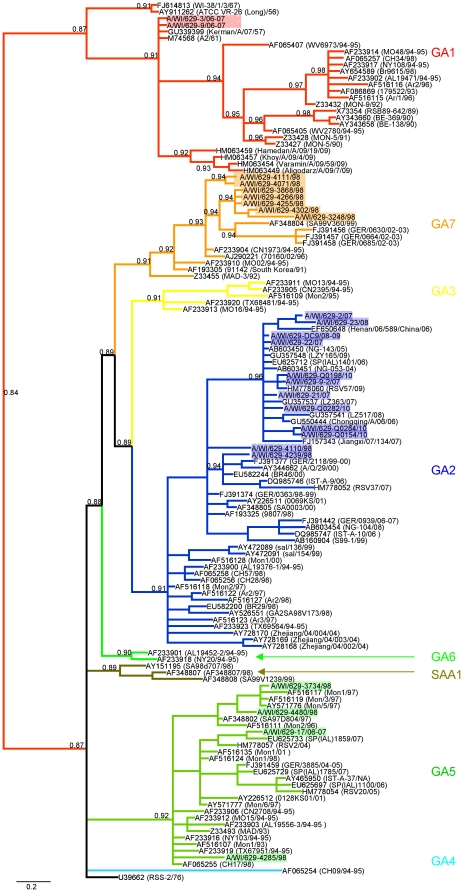
Phylogenetic tree of the highly variable C-terminal domain of the G protein CDS of RSV A. Strains worldwide already published are identified with the GenBank identification number and in parenthesis are the correspondent published strain names with the last two numbers of the year of collection and where NA denotes that the collection date was not available. The 25 sequences from the Milwaukee strains are highlighted. The tree was calculated using 1×10^6^ generations and a burn in of 50 in MrBayes. Node values correspond to posterior probabilities.

**Figure 4:**
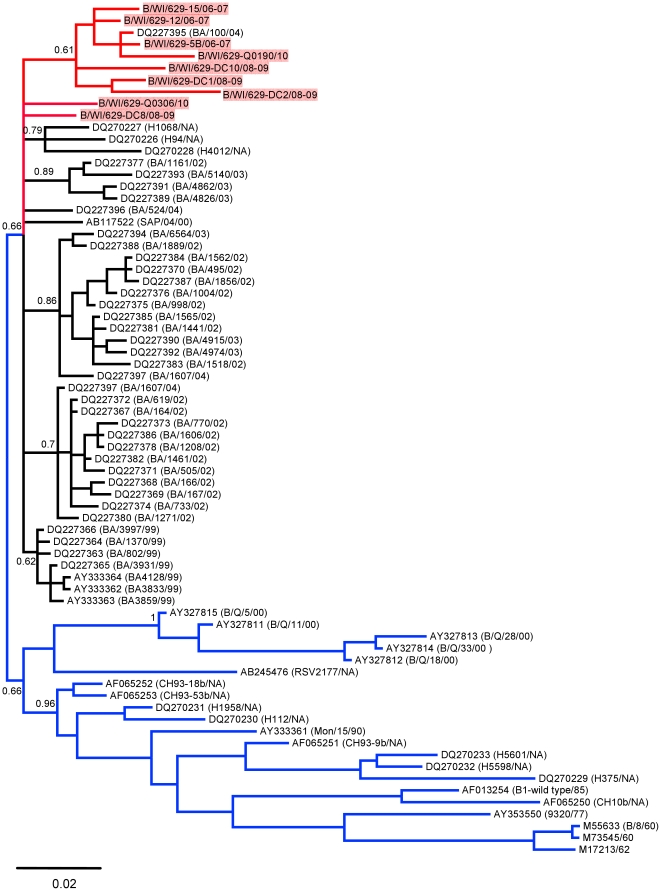
Phylogenetic tree of the whole G protein CDS of RSV B. The sequences from Milwaukee are colored and highlighted in red and the sequences from non-BA genotypes are colored in blue. Node values correspond to posterior probabilities. The tree was calculated using 3×10^6^ generations and a burn in of 170 in MrBayes. (In parenthesis are the published strain names). Strains worldwide already published are identified with the GenBank identification number and in parenthesis are the correspondent published strain names with the last two numbers of the year of collection and where NA denotes that the collection date was not available.

RSV A and RSV B sequences were analyzed by neighbor-joining, maximum parsimony and Bayesian phylogeny methods. The topography of the trees for the individual CDSs generally agreed with the trees of the whole genomes for RSV A and RSV B (Figures 1 and 2). All three methods produced the same phylogenies showing well supported tree topologies. Likewise, the phylogenetic trees for the G protein CDS and its highly variable C-terminal domain had very similar topology for RSV A (Figure 3) and RSV B (Figure 4).

All RSV A sequences grouped with previously assigned genotypes, GA1, GA2, GA5 and GA7 (Figures 1 and 3). This shows a high diversity in the Milwaukee area where these genotypes co-circulated during the 1997–1998 and 2006–2007 seasons, and genotype GA2 persisting during the last 13 years (from 1998–2010) and genotype GA5 persisting at least for 11 years (during 1996–2007). Previous studies reported the same stability of the genotype GA2 for more than 20 years in Sweden during 1976–2004 [28], and 7 years in Argentina during 1997–2004 [29]. Genotype GA1 was considerably divergent from the other genotypes as shown by other authors before [7]. Interestingly, two strains (A/WI/629-3/06-07 and A/WI/629-9/06-07) isolated in Milwaukee grouped together in GA1 with the prototype strain A2 isolated in 1961. Temporal and geographical clustering have been previously described for RSV worldwide [7], [8], [29]–[33]. Most notably, GA2 shows sub-clades corresponding with the year of collection suggesting a temporal relationship rather than geographical (Figures 1 and 3). Genotype GA2 is the most geographically diverse containing sequences from USA, China, Japan, Brazil, Uruguay, Argentina, Ireland, Germany, Istanbul, and South Africa (Figure 3).

For RSV B the neighbor-joining and maximum parsimony trees generally supported the topology of the Bayesian trees with slight variations primarily in the shorter CDSs (SH and M2-2) which did not have enough variation to achieve adequate resolution (data not shown). The phylogenetic tree for the RSV B genomes show that all of the sequences from our local strains have little variation and appear to all be from a single clade. This is supported by the trees for the G protein CDS and C-terminal domain in which the Milwaukee sequences cluster separately from the majority of the published sequences for this CDS. Most of the individual CDS trees had lower resolution due to the low amount of overall variation in RSV B (not shown). All of our RSV B genome sequences had significantly less variation between each other than they did with the two published genomes. A tree of the G gene revealed that all of RSV B sequences from this study were in a separate clade from all of the published sequences except for one strain named BA/100/04 (DQ227395) which was collected in Argentina in 2004 (Figure 4). This strain was sequenced as part of a study of 38 BA genotype RSV B strains in which the entire G protein CDS had been sequenced, 35 of which are present in our tree (Figure 4) [10]. In that study BA/100/04 was classified as belonging to the BA-IV subgroup. Not surprisingly, there was also clear separation between the BA and non-BA genotypes in both trees.

### Synonymous and Non-synonymous Mutations

The data from SNAP shows that for all CDSs there was more variation in RSV A than RSV B sequences (Table 2). However, the number of non-synonymous mutations was slightly higher in some of the RSV B CDSs (NS1, NS2, M, and M2-1). The CDSs with the highest dN/dS values were those for the G, M2-2, and SH CDSs. Interestingly, the fact that the SH, G, and M2-2 CDSs have the highest ratios of non-synonymous mutations within each subgroup correlates with the fact that these three proteins also have the lowest amino acid identity between RSV A and B [34]. In RSV B 59% of the variable sites are accounted for by differences between the published sequences from strains that date back more than 26 years and our sequences (2006–2010). The RSV A genome analysis demonstrated that only 10% of the observed variation comes from the difference between our isolates and previously published sequences.

**Table 2:** Variation of each coding sequence in RSV A and B.

Gene	RSV A					RSVB				
	Average d_S_	Average d_N_	d_N_/d_S_	Total Sites	Variable Sites	Average d_S_	Average d_N_	d_N_/d_S_	Total Sites	Variable Sites
NS1	0.1285	0.0022	0.017	420	42	0.0478	0.0044	0.092	420	20
NS2	0.1224	0.0037	0.030	375	37	0.0710	0.0037	0.052	375	25
N	0.1229	0.0018	0.015	1176	117	0.0389	0.0008	0.019	1176	42
P	0.0876	0.0034	0.039	726	57	0.0710	0.0028	0.040	726	45
M	0.1008	0.0008	0.008	771	59	0.0432	0.0013	0.031	771	34
SH	0.1123	0.0052	0.046	195	19	0.0258	0.0045	0.173	198	7
G	0.1041	0.0478	0.460	897	202	0.0520	0.0195	0.376	939	111
F	0.1196	0.0058	0.049	1725	190	0.0598	0.0024	0.040	1725	88
M2-1	0.1021	0.0056	0.054	585	55	0.0745	0.0064	0.086	588	38
M2-2	0.0585	0.0286	0.488	267	33	0.0517	0.0080	0.155	273	16
L	0.1042	0.0044	0.042	6498	593	0.0494	0.0021	0.042	6501	287

### Genotype divergence

The dS/dN rate has been used as an indicator of selective pressure with dS/dN = 1 meaning neutral mutation, dS/dN>1 meaning purifying selection or negative selection, and dS/dN<1 meaning positive selection [35]. GA2 has an average dS/dN of 1.025 which indicates the most neutral selection while the other genotypes GA1 (1.23), GA7 (1.28) and GA5 (2.31) showed signs of purifying selection.

### Intergenic Sequence Analysis

The intergenic sequences (IGS) vary in length (1–52 nt) in RSV A, and (3–55 nt) in RSV B. Comparing each of the IGS of the strains to the correspondent IGS of the prototype A2 strain found that two strains (A/WI/629-3/06-07 and A/WI/629-9/06-07) have identical sequences for all eight IGS analyzed. The average identity to A2 calculated for all IGS ranged from 33% for P/M (due to a five nt deletion) to 89% for NS1/NS2 and F/M2 (Table 3).

**Table 3:** Lengths and conservation of the NCR and IGS regions in RSV A and B.

Gene	RSV A						RSV B					
	5′NCR Length^a^	Conservation (%)	3′NCR Length	Conservation (%)	Intergenic Length	Conservation (%)	5′NCR Length^a^	Conservation (%)	3′NCR Length	Conservation (%)	Intergenic Length	Conservation (%)
**NS1**	45	95.56	45	95.56	19	89.47	45	95.56	45	95.56	17	100
**NS2**	23	91.3	73	89.04	26	84.62	23	86.96	85	92.94	26 (29)	88.46
**N**	6	83.33	0	-	1 (2)^b^	100	6	100	0	-	3	100
**P**	8	100	156 (155)	84.62	9 (4,5)	33	8	100	158	94.94	9	100
**M**	0	-	165	83.03	9 (8)	77.78	0	-	162	89.51	9	88.89
**SH**	75	78.67	121	76.86	44	86.36	76	90.79	117	87.18	44	86.36
**G**	6	100	61	73.77	52	88.46	6	100	19	94.74	52	98.08
**F**	4	100	65	87.69	46	89.13	4	100	150 (151)	93.33	55	94.55
**M2**	0	-	-	-	-	-	0	-	-	-	-	-
**L**	-	-	60 (54,61)	90	-	-	-	-	58	98.28	-	-

a Lengths do not include GS, GE, start codon, or stop codon sequences.

Values in parentheses indicate alternate lengths observed, excluding those limited to previously published sequences since they cannot be verified.

In general, the Milwaukee strains belonging to the same genotype had intergenic regions that were more similar to each other than to another genotype or to A2 as described previously [36]. Additionally, the M/SH, G/F, and F/M2 sequences presented a conserved pattern characteristic of each of the four genotypes described for the Milwaukee strains (Table 4). The two strains with identical IGS to A2 described above belong to the same genotype GA1 and we observed that the IGS of GA1>GA7>GA5>GA2 exhibit the highest to the lowest identity to A2. RSV B strains showed IGS identity to the prototype strain 9320/77 between 86–91% which were comparable to the IGS for RSV A.

**Table 4:** Intergenic region sequences for RSV A strains from Milwaukee and genotype patterns.

Intergenic regions	Sequence 3′→5′ a	Genotype
**SH-G (44)**	UAGUCAUAACAAUGAACUAGGAUAUCAAGACUAACAAUAACAUU	GA1
	.........................U.....C..A..C..UGC.	GA2 (2010)
	................U........U.....C..A..C...G..	GA2 (2007–2009)
	...................A.....U.....C.....C...G..	GA2 (1998)
	...................A.....U...........C...G..	GA5
	...................AA....U...............G..	GA7
**G-F (52)**	CAUAUUAUCACAAAAAGCCAUGACCAACUUAAACAGAAUCAAAAUAAACUCU	GA1
	.G....G.UG.................A.C.....U.........C......	GA2
	.G......UG......A..........A...................G....	GA5
	.G......UG.................A.C...............C......	GA7
**F-M2 (48)**	CACAAUUGAAUGCCAGAUUAACUUACCAUCUGUAAAAAUGAAAACUG	GA1
	-..........A...A..........U..U...........G.....	GA2
	-..........A..............U..U...........G.....	GA5
	-..........A..............U..U.................	GA7

a After alignment of the IGS of the prototype strain A2 with the IGS of the Milwaukee strains, a comparison was made to find out the identical/variable nucleotides. Identical nucleotides are represented by a “.” while variable nucleotides are represented by their nucleotide difference when compared to the prototype strain. These differences are what make up the genotype-specific patterns.

### Gene start and end sequences

All *Paramyxoviruses* have been shown to contain conserved gene start (GS) and gene end (GE) sequences [6]. For RSV A all of the GS and GE sequences have been described and analyses have been performed to identify the functional importance of differences within and around these sequences [36]–[40]. To our knowledge the GS and GE sequences have only been described for 7 of the genes (NS1, NS2, N, M, G, F, and M2) in RSV B despite the existence of two complete wild-type genomes for this subgroup [41] and no functional studies have been performed. The remaining GS and GE sequences for RSV B were identified based on their positional and sequence similarity to their respective sequences in RSV A. In both RSV A and B the GS consensus sequence is 3′-CCCCGUUUA-5′ with the only exception being the L gene in both RSV A and B (3′-CCCUGUUUU-5′) and the SH gene in RSV B (3′-CCCCAUUUA-5′) (Table 5). The GS sequences were 100% conserved in all of the strains we sequenced for both RSV A and B.

**Table 5:** The gene end sequences of RSV A and B.

Gene	RSV A		RSV B	
	Gene End Sequences 3′→5′^a^	Number of Strains^b^	Gene End Sequences 3′→5′	Number of Strains
**NS1**	UCAAUUAUAUUUU (A2)	24	UCAAUUAUAUUUUU (9320)	0
			**UCAAUUAUAUUUU**	9
**NS2**	UCAUU-AAAUUUU (A2)	13	UCAUU–AAUUUUU (9320)	9
	**UCAAU-AAAUUUU**	12		
**N**	UCAAUU–AUUUUUU (A2)	17	UCAAUU–GUUUUUU (9320)	9
	UCAAUU–AUUUUUUU (19)	4		
	UCAAUU–AUUUUUUUU (S2)	0		
	**UCAUU–AUUUUUU**	1		
**P**	UCAAU–GUUUUUUU (A2)	23	UCAUU–GUUUUUUU (9320)	9
	UCAAU–GUUUUUU	1		
	**UCAAU–GAUUUUUU**	1		
**M**	UCAAUU–AUUUUUU^c^ (A2)	12	UCCAUU-UAUUUU (9320)	9
	UCAAUU–AUUUUU (Long,S2)	13		
	**UCAAUU–AUUUUUUU** c	1		
	**UCAAUU-ACUUUUU**	1		
**SH**	UCAAUU-AAUUUUU (A2)	17	UCAAU-AAAUUUU (9320)	7
	UCAAUU-AAUUUUUU	7	**UCAAU-AAAUUUUU**	2
	**UCAAUU-AAUUUUUUU**	1		
**G**	UCAAU-GAAUUUUU (A2)	2	UCAAU-AAGUUUUU (9320)	0
	UCAAU–AAUUUUUU (Long,19)	0	**UCAAU-AAAUUUUU**	7
	UCAGU–AAUUUUU (S2)	23		
**F**	UCAAU-AUAUUUU (A2)	20	UCAAU-GUAUUUU (9320)	8
	UCAAU-AUAUUUUU	4		
**M2**	UCAAU-AAAUUUU (A2)	23	UCAAU-AGAUUUU (9320)	7
			UCAAU-AAAUUUU (B1)	0
**L**	UCAAU–AAUUUUU (A2)	2	UCAAU–AAUUUUU (9320)	8
	UCAAU–AAUUUUUU (Long,19)	0	**UCAAU–AAUUUU**	1
	UCAAU-AAAUUUU	23		

a Bold sequences have not been previously identified by Moudy 2003 or in published genomes. Dash characters have been added to the central domain so that the U-tracts line up. Sequences that are found in the published genomes are marked with that strain in parentheses. Genomes other than A2 for RSV A and 9320 for RSV B are only listed if they have a different GE sequence.

b These numbers only include strains sequenced in this study.

c 629-Q0198 is a mix of 6 and 7 U's but was only added to the GE with 6 U's.

In RSV A the GE consensus has been described as (3′ - UCAAUN1–4U4–7 - 5′) and it has been suggested that it should include an “A” at the 3′ end which is conserved in 8 of the 10 GE sequences in RSV A [6] and 9 of the 10 GE sequences in RSV B (Table 5). Most variation has been found in the central unconserved region and in the length of the U tract while the UCAAU region is largely conserved, however, there are some notable exceptions. The most common variation of this region is UCAUU and has been identified in the NS2 GE in both RSV A and B, the N GE of RSV A and the P GE of RSV B. Another, variation (UCAGU) has been shown to be present in the G GE for all RSV A strains that don't belong to the GA1 genotype [36]. This includes 23 of the strains sequenced in our study. For the M GE in RSV B all strains have UCCAU for this region. All other GE sequences observed for RSV A and B matched the consensus sequence. Between what was published previously and the sequences in this study the only GE sequence that is completely conserved in all sequences for RSV A is that of the M2 gene [36]. For RSV B the GE sequences for the NS1, N, P, M, and F genes were conserved in both the sequences published in this study and the two published whole genomes. Between RSV A and B only 3 genes (NS1, M2, and L) had a GE variant that was shared between both groups. For all other genes there were one or two differences between GE sequences.

### Non-coding Regions Analysis

**Figure 5:**
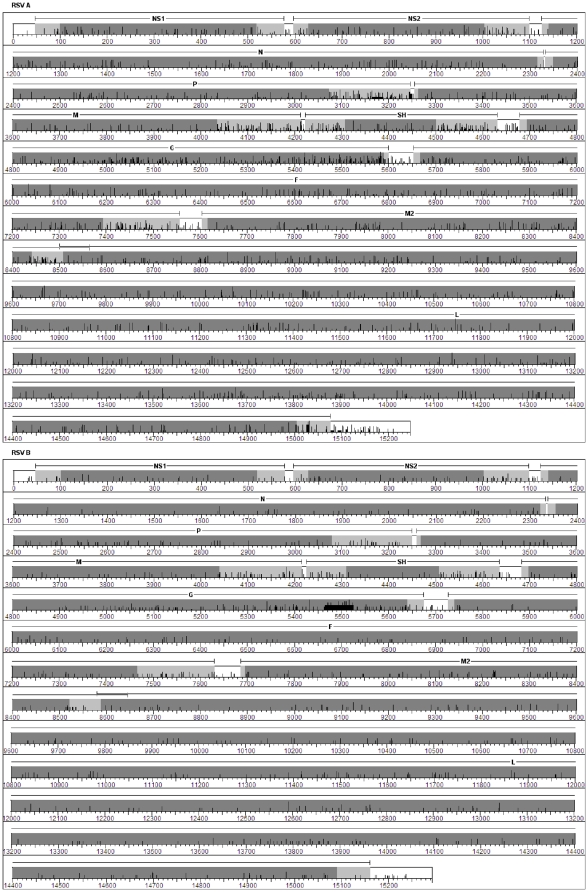
Entropy plot and graphical representations of the RSV A and B genomes . The entropy data is represented by the vertical black bars with the scale for the y-axis being from 0 to 1. The higher the bar the more variation (mutations, insertions, or deletions) at that position in the genome alignments. The dark grey bars represent the coding regions. The M2-1 and M2-2 coding regions overlap so are represented by a single bar. The light grey regions correspond to the 5′ and 3′ NCRs of each gene and include the GS and GE signals. The white regions correspond to the non-genic regions of the genome. Above the plots, each gene is labeled and there is a bar that runs from the beginning to end of each gene.

The analysis of the 5′NCRs and 3′NCRs for each RSV gene (Table 3) shows that the 5′NCRs are generally shorter than the 3′NCRs for each gene and the short ones (4–8 bp) are 100% conserved in RSV group A and B. In general, the 3′NCRs presented more variation than the 5′NCRs for each gene and the most variable ones are present in: the G gene for RSV A, the M gene for RSV B, and in the SH gene for RSV A and B (Table 3), (Figure 5). The 3′NCRs in RSV A were 4–22% more variable than the 3′NCRs in RSV B, with the highest variation observed in the G gene. 5′NCRs and 3′NCRs were 100% conserved for sequences belonging to GA1 and the most variable 3′NCRs were found in sequences of the G gene belonging to GA2 and GA7 (data not shown). Genotype-specific patterns were observed for most of the NCR in RSV A especially for NS1, NS2, P and the 3′NCR of the L gene. Patterns were not observed in short NCRs. For RSV B we could not find any pattern characteristic of genotype due to the lack of genetic variation in this subtype.

### Glycoprotein Gene Insertions and Deletions

For the glycoprotein (G) gene we identified three RSV strains that had mutations which should cause pre-mature stop codons. RSV A strain 629–4285 has a 2 bp mutation that changed codon 212 from AAA to TGA and is predicted to cause the G protein to be truncated to 211 amino acids. Two RSV B strains (629-12 and 629-DC10) were identified to contain a single nucleotide deletion within the glycoprotein CDS. In 629-12 this deletion occurred at position 582 of the CDS and caused a pre-mature stop codon at amino acid position 198 of the G protein which would otherwise be 310 amino acids long. For 629-DC10 the deletion occurred at nucleotide position 690 which would cause a stop codon at amino acid 233. Analysis of the chromatograms for this region suggest that in this sample there is actually a mixed population of RSV in which some virus particles contain this deletion and some do not. While not necessarily related, we did attempt to isolate 629–4285 and 629-DC10 in tissue culture, but were unsuccessful. Since we were not able to isolate these strains and did not have enough material to do more testing we could not verify their viability. It has previously been shown *in vitro* that RSV mutants lacking the G gene are still able to form infectious particles and replicate although less efficiently [42], it is possible to generate RSV strains with truncated G proteins *in vitro* using sub-neutralizing levels antibody [43], and RSV with a truncated G protein has even been isolated from an immunocompromised patient [44]. Therefore, it is certainly possible that an individual could develop a respiratory-tract infection with a strain of RSV containing a truncated version of the G protein.

Also, all of the Milwaukee RSV B sequences contained the 60 bp insertion (positions 5465–5525 in Figure 5) characteristic of the BA genotype and a 6 bp deletion between positions 470–480 relative to the reference sequences. The exact 6 bp deleted cannot be determined due to repetition in this region. This same deletion was present in three other published sequences (DQ227395, DQ270232, and DQ270233). Interestingly DQ270232 and DQ270233 do not belong to the BA genotype and this deletion was not present in the majority of published BA genotype sequences which suggests that this deletion has occurred on at least two separate occasions [45].

## Discussion

We report the successful development of a rapid strategy for whole genome sequencing for RSV and the results of applying it to 34 RSV positive clinical samples collected over a 13 year time period. In addition we have reported the analysis of this genomic data and comparison to all relevant RSV genomic data available in GenBank.

Significant diversity among RSV A Milwaukee strains was observed and can be explained by the co-circulation of multiple genotypes within and across seasons. GA2 and GA5 co-circulated in two different seasons (1998 and 2006) after which apparently only GA2 persisted, still infecting people in the Milwaukee area in 2010. These results are consistent with other published studies for RSV A [29], [46] where they also had shown that several genotypes are present now, were present in 1996, and were even present much earlier as can be seen by the fact that published sequences from as far back as 1956 fall into these same genotypes (Figure 3). These genotypes appear to have much more distant origins and continue to co-circulate with each genotype steadily accumulating unique mutations. So the significant amount of variation in RSV A is primarily due to the presence of co-circulating genotypes rather than the rapid accumulation of mutations over a short period of time.

A similar situation can be described in our community with the strains A/WI/629-3/06-07 and A/WI/629-9/06-07 isolated during the 2006/2007 season and which belong to the same lineage (GA1) as the prototype strain A2 isolated almost 50 years ago (Figures 1 and 3). These two strains show surprising similarity to the published A2 sequence (M74568) with only 15 and 19 mutations respectively, but they are actually more similar to mutant strains of the virus (U50362, U50363, and U63644). We do have the A2 ATCC strain (VR-1540) in our lab so there is some possibility of contamination with this strain in these samples even though the ATCC strain was not used in this study or in use when sequencing these strains. Since A/WI/629-9/06-07 was sequenced directly from a clinical sample and has the most mutations compared to the published A2 sequence contamination with the ATCC strain or A/WI/629-3/06-07 seems unlikely. Another possibility is that the A2 strain may have been accidentally re-introduced into the population through exposure of laboratory workers, which could also explain the relatively few mutations. Furthermore, although GA1 has not been observed in the last 15 years in European countries, South America or North America, it has been detected again in the last 4 years not only in Milwaukee but also in South Asia as reported recently (GenBank: HM063457, HM063454, GU339399). It is interesting to see that this genotype may have been re-introduced into North America or continued circulating at least until 2007 in Milwaukee. Unfortunately, the lack of molecular epidemiological studies in the USA for the last 12 years makes it difficult to determine whether this lineage has reemerged or has been continuously circulating during this time.

Other authors demonstrated that the continuous circulation of strains belonging to the genotype GA2 worldwide, and for which more amino acid changes were observed, compared with strains belonging to other genotypes, is due to positive selection pressure [47]–[49]. Although the limited number of samples sequenced in this study per year precludes definitive conclusions about the periods of circulation of different genotypes in Milwaukee, the more amino acid changes observed in GA2 compared with other genotypes correlate with the continuous and potentially longer circulation of GA2 relative to other genotypes noted in our community. A more accurate picture of the persistence and co-circulation of other genotypes could have been determined with the inclusion of more strains.

The C-terminal region of the G-protein has been described as the most variable one between and within RSV group A and B and has been designated as the most reliable to describe the evolutionary changes of RSV [7], [50]. Since Peret *et al.* reported in 1998 for the first time the existence of different genotypes based on the analysis of this short C-terminal part of the G gene, the majority of the evolutionary and molecular studies carried out on RSV A and B have been focused on this region [7]. To date, our knowledge on the molecular evolution of RSV is mainly based on studies done using the C-terminal region, which just accounts for 2% of the whole RSV genome. In this report we were able to independently confirm this hypothesis using the entire RSV genome and demonstrate that the same evolutionary pattern and clades were seen with the whole genome as with the G protein CDS and its C-terminal. Interestingly, we also observed genotype-specific patterns throughout other parts of the RSV A genome namely the IG sequences and the NCRs revealing that the nucleotide phylogeny of RSV A is consistent across its whole genome. This particular characteristic suggests that not only the commonly used C-terminal of the G gene allows for genotyping but also that other parts of the genome are important to describe evolution in RSV A as well. Since the C-terminus of the G protein CDS is still the most variable allowing for the best discrimination between strains and has been used by the majority of the evolutionary studies it is recommended to continue to use this region for genotyping when larger portions of the genome are incapable of being sequenced so as to maintain comparability between studies.

The situation was quite different for RSV B. Previous studies identified a new genotype of RSV B which contained a 60 nt duplication in the C-terminus of the glycoprotein gene. This genotype was first identified in samples collected in Buenos Aires during the 1999 RSV season and given the designation BA [11]. Later studies identified this genotype in samples collected in Spain and Belgium also during 1999 and in one sample collected in December of 1998 [12], [51]. Since the BA genotype was already present in both South America and Europe in 1999 and there was little variation between the sections of the G gene sequenced in these studies, it is believed that the 60 bp insertion event must have occurred no more than one or two years earlier. However, since no studies have yet identified BA strains in earlier seasons, it is possible that the first occurrence was in Europe during the 1998/1999 season. Multiple studies have demonstrated that since this insertion first occurred the BA genotype has spread worldwide and become the dominant RSV B genotype [10], [12], [51], [52]. Therefore, it is not surprising that all of the RSV B strains identified in our study (2006–2010) belong to the BA genotype. Since all of the RSV B sequences in our study are from this relatively recent genotype it is also not surprising that we saw a small amount of variation between sequences with most of the total variation observed coming from differences between our sequences and the previously published RSV B genome sequences. While the data from our study for the CDS regions, GS and GE sequences, 5′ and 3′NCRs, and IGS all suggest that there is less variation in RSV B than RSV A, a previous study looking at a 724 bp region of the G gene for 196 RSV B strains from 22 seasons (1982–2004) found that the evolutionary rates for this gene were very similar to that observed in RSV A [12]. This discrepancy is most likely due to our limited data set and it would therefore be important to do a larger study for RSV B incorporating strains from other genotypes that occurred in previous seasons.

When comparing all eight IGS of the Milwaukee strains with the prototype strains for RSV A and RSV B (GenBank: M74568 and AY353550), very low variability over the last 50 and 34 years respectively was observed. The degree of variation in the intergenic sequences seen for the RSV Milwaukee strains group A and group B agreed with previous studies on RSV A clinical isolates from the USA and UK [36]. Unlike some *Paramyxoviruses* that present conserved or semi-conserved di- or trinucleotide IGS patterns [34], [53]–[55] RSV shows longer and more variable IGS regions.

In RSV, transcription starts at a single promoter located at the 3′ end of the genome. After binding to that promoter the polymerase scans towards the 5′ end of the genome until it reaches a specific GS sequence which initiates transcription of the mRNA for each gene. The polymerase continues to produce the mRNA until it reaches a GE sequence. Depending on the sequence of the GE it will either stop producing mRNA (terminate) or continue through the next gene until it reaches another GE sequence producing a dicistronic mRNA [6], [38] Because transcription is always initiated at the 3′ end of the genome and some percentage of the polymerase falls off at each GE sequence the number of mRNAs transcribed for each gene decreases proportionately from the 3′ end to the 5′ end of the genome.[36]. This in turn leads to decreased protein produced for genes at the 5′ end of the genome [36], [56]. It is in this manner that the termination efficiency of the GE seems to be used by RSV to regulate the production of its proteins. Multiple studies have shown that the transcription termination efficiency is principally regulated by the specific GE sequence with a little influence from individual bases of the 3′NCR and IGS of a gene [36]–[38], [40], [56]. The GE of the RSV A SH gene has been described as the ideal termination sequence and its high termination efficiency is evidenced by the lack of detectable SH-G dicistronic mRNA in cell culture. Additional studies have directly measured its termination efficiency [6], [40]. Detailed mutational studies have been performed with GE sequences from RSV A identifying that different mutations in various individual positions or combinations of positions can have a significant impact on termination efficiency [36], [38], [39]. Mutations in the UCAAU region in particular caused a significant decrease in termination efficiency. Since the GE sequences between RSV A and B are similar it is likely variations in RSV B GE sequences would have a similar impact on termination efficiencies. For example, since the matrix gene GE in all RSV B sequences contained an A->C mutation in the UCAAU region it is possible that termination efficiency is reduced at this location which could result in a decrease in the production of the SH protein during RSV B infection compared to that of RSV A. The additional variations that we identified for RSV A further highlight just how variable these GE sequences are. The most common variation that we saw was a change in the length of the U-tract. It has previously been shown that longer U-tracts have increased termination efficiency and are less sensitive to mutations at other positions in the GE sequence [6], [36]. Interestingly for RSV B we saw less variation within GE sequences for each individual gene with half of them showing no variations and the remaining genes only having two variants each. It is possible that RSV B is more sensitive to changes in termination efficiency so that there is more selective pressure preventing changes in the GE sequences. However, the lack of variation could also just be an artifact of small sample size primarily from a single genotype that is relatively young. More sequences from older RSV B non-BA genotypes would provide a more accurate picture of the true RSV B GE diversity.

RSV is a health concern worldwide causing significant morbidity, mortality and cost to our society [57]. The sequencing method presented in this work allows four RSV genomes to be sequenced simultaneously in as little as two working days. The low cost and ability to perform this assay on clinical specimens could increase its use as a routine clinical assay. Our study significantly increased the amount of genomic data available for this important respiratory pathogen. With this additional genomic data we identified variable regions throughout the genome that may be beneficial for evolutionary studies and conserved regions that will be beneficial for the development of improved diagnostics. While our study is limited to strains from Milwaukee collected during the last 14 years, the data from this study will be beneficial to comprehensive studies of RSV worldwide. These whole genome sequences will benefit other communities by eliminating the dependence on the limited information contained in the tiny fractions of the RSV genome previously published. Our data contributes to a better knowledge of the basic molecular structure of RSV, which will help drive the development of new vaccine approaches and allow for control and reduction of the disease burden caused by RSV.

## Supporting Information

Figure S1 **RNA negative-sense genome and amplification strategy of the whole genome of RSV.** At the top the kilobase scale is aligned relative to the RSV genome (approximately to scale). Genes are represented as green rectangles named according to the encoded protein. At the bottom the 12 PCR products resulted from the cDNA amplification with 12 overlapping primer sets spanning the entire RSV genome.
(TIF)

Table S1 **Primers used for amplification and sequencing.**
(DOC)

## References

[1] Harish Nair, Nokes DJ, Gessner BD, Dherani M, Madhi SA (2010). Global burden of acute lower respiratory infections due to respiratory syncytial virus in young children: a systematic review and meta-analysis.. Lancet 2010; published online April.

[2] UN Population Division. World population prospects: the 2008 revision.. http://esa.un.org/unpp.

[3] Nokes JD, Cane PA (2008). New strategies for control of respiratory syncytial virus infection.. Curr Opin Infect Dis.

[4] Anderson LJ, Hierholzer JC, Tsou C, Hendry RM, Fernie BF (1985). Antigenic characterization of respiratory syncytial virus strains with monoclonal antibodies.. J Infect Dis.

[5] Mufson MA, Orvell C, Rafnar B, Norrby E (1985). Two distinct subtypes of human respiratory syncytial virus.. J Gen Virol.

[6] Knipe DM, Howley PM, Kluwer W (2007). Respiratory Syncytial Virus and Metapneumovirus.. Fields Virology.

[7] Peret TC, Hall CB, Schnabel KC, Golub JA, Anderson LJ (1998). Circulation patterns of genetically distinct group A and B strains of human respiratory syncytial virus in a community.. J Gen Virol.

[8] Peret TC, Hall CB, Hammond GW, Piedra PA, Storch GA (2000). Circulation patterns of group A and B human respiratory syncytial virus genotypes in 5 communities in North America.. J Infect Dis.

[9] Venter M, Madhi SA, Tiemessen CT, Schoub BD (2001). Genetic diversity and molecular epidemiology of respiratory syncytial virus over four consecutive seasons in South Africa: identification of new subgroup A and B genotypes.. J Gen Virol.

[10] Trento A, Viegas M, Galiano M, Videla C, Carballal G (2006). Natural history of human respiratory syncytial virus inferred from phylogenetic analysis of the attachment (G) glycoprotein with a 60-nucleotide duplication.. J Virol.

[11] Trento A, Galiano M, Videla C, Carballal G, Garcia-Barreno B (2003). Major changes in the G protein of human respiratory syncytial virus isolates introduced by a duplication of 60 nucleotides.. J Gen Virol.

[12] Zlateva KT, Lemey P, Moes E, Vandamme AM, Van Ranst M (2005). Genetic variability and molecular evolution of the human respiratory syncytial virus subgroup B attachment G protein.. J Virol.

[13] Sato M, Saito R, Sakai T, Sano Y, Nishikawa M (2005). Molecular epidemiology of respiratory syncytial virus infections among children with acute respiratory symptoms in a community over three seasons.. J Clin Microbiol.

[14] Dapat IC, Shobugawa Y, Sano Y, Saito R, Sasaki A (2010). New genotypes within respiratory syncytial virus group B genotype BA in Niigata, Japan.. J Clin Microbiol.

[15] Shobugawa Y, Saito R, Sano Y, Zaraket H, Suzuki Y (2009). Emerging genotypes of human respiratory syncytial virus subgroup A among patients in Japan.. J Clin Microbiol.

[16] Zlateva KT, Vijgen L, Dekeersmaeker N, Naranjo C, Van Ranst M (2007). Subgroup prevalence and genotype circulation patterns of human respiratory syncytial virus in Belgium during ten successive epidemic seasons.. J Clin Microbiol.

[17] Zhang ZY, Du LN, Chen X, Zhao Y, Liu EM (2010). Genetic variability of respiratory syncytial viruses (RSV) prevalent in Southwestern China from 2006 to 2009: emergence of subgroup B and A RSV as dominant strains.. J Clin Microbiol.

[18] Tolley KP, Marriott AC, Simpson A, Plows DJ, Matthews DA (1996). Identification of mutations contributing to the reduced virulence of a modified strain of respiratory syncytial virus.. Vaccine.

[19] Crowe JE, Bui PT, Firestone CY, Connors M, Elkins WR (1996). Live subgroup B respiratory syncytial virus vaccines that are attenuated, genetically stable, and immunogenic in rodents and nonhuman primates.. J Infect Dis.

[20] Beck ET, Jurgens LA, Kehl SC, Bose ME, Patitucci T (2010). Development of a rapid automated influenza A, influenza B, and respiratory syncytial virus A/B multiplex real-time RT-PCR assay and its use during the 2009 H1N1 swine-origin influenza virus epidemic in Milwaukee, Wisconsin.. J Mol Diagn.

[21] Kehl SC, Henrickson KJ, Hua W, Fan J (2001). Evaluation of the Hexaplex assay for detection of respiratory viruses in children.. J Clin Microbiol.

[22] Katoh K, Kuma K, Toh H, Miyata T (2005). MAFFT version 5: improvement in accuracy of multiple sequence alignment.. Nucleic Acids Res.

[23] Hall TA (1999). BioEdit: a user-friendly biological sequence alignment editor and analysis program for Windows 95/98/NT.. Nucl Acids Symp Ser.

[24] Huelsenbeck JP, Ronquist F (2001). MRBAYES: Bayesian inference of phylogenetic trees.. Bioinformatics.

[25] Swofford DL (1998). Phylogenetic Analysis Using Parsimony (and Other Methods); Sinauer Associates S, editor..

[26] Nei M, Gojobori T (1986). Simple methods for estimating the numbers of synonymous and nonsynonymous nucleotide substitutions.. Mol Biol Evol.

[27] Korber B (2000). HIV Signature and Sequence Variation Analysis.. Computational Analysis of HIV Molecular Sequences.

[28] Ostlund MR, Lindell AT, Stenler S, Riedel HM, Wirgart BZ (2008). Molecular epidemiology and genetic variability of respiratory syncytial virus (RSV) in Stockholm, 2002-2003.. J Med Virol.

[29] Viegas M, Mistchenko AS (2005). Molecular epidemiology of human respiratory syncytial virus subgroup A over a six-year period (1999-2004) in Argentina.. J Med Virol.

[30] Choi EH, Lee HJ (2000). Genetic diversity and molecular epidemiology of the G protein of subgroups A and B of respiratory syncytial viruses isolated over 9 consecutive epidemics in Korea.. J Infect Dis.

[31] Scott PD, Ochola R, Ngama M, Okiro EA, Nokes DJ (2004). Molecular epidemiology of respiratory syncytial virus in Kilifi district, Kenya.. J Med Virol.

[32] Kuroiwa Y, Nagai K, Okita L, Yui I, Kase T (2005). A phylogenetic study of human respiratory syncytial viruses group A and B strains isolated in two cities in Japan from 1980-2002.. J Med Virol.

[33] Matheson JW, Rich FJ, Cohet C, Grimwood K, Huang QS (2006). Distinct patterns of evolution between respiratory syncytial virus subgroups A and B from New Zealand isolates collected over thirty-seven years.. J Med Virol.

[34] Lamb RA, Kolakofsky D (1996). Paramyxoviridae: the viruses and their replication..

[35] Miyata T, Yasunaga T (1980). Molecular evolution of mRNA: a method for estimating evolutionary rates of synonymous and amino acid substitutions from homologous nucleotide sequences and its application.. J Mol Evol.

[36] Moudy RM, Sullender WM, Wertz GW (2004). Variations in intergenic region sequences of Human respiratory syncytial virus clinical isolates: analysis of effects on transcriptional regulation.. Virology.

[37] Harmon SB, Wertz GW (2002). Transcriptional termination modulated by nucleotides outside the characterized gene end sequence of respiratory syncytial virus.. Virology.

[38] Kuo L, Fearns R, Collins PL (1997). Analysis of the gene start and gene end signals of human respiratory syncytial virus: quasi-templated initiation at position 1 of the encoded mRNA.. J Virol.

[39] Sutherland KA, Collins PL, Peeples ME (2001). Synergistic effects of gene-end signal mutations and the M2-1 protein on transcription termination by respiratory syncytial virus.. Virology.

[40] Hardy RW, Harmon SB, Wertz GW (1999). Diverse gene junctions of respiratory syncytial virus modulate the efficiency of transcription termination and respond differently to M2-mediated antitermination.. J Virol.

[41] Johnson PR, Collins PL (1988). The A and B subgroups of human respiratory syncytial virus: comparison of intergenic and gene-overlap sequences.. J Gen Virol.

[42] Techaarpornkul S, Barretto N, Peeples ME (2001). Functional analysis of recombinant respiratory syncytial virus deletion mutants lacking the small hydrophobic and/or attachment glycoprotein gene.. J Virol.

[43] Rueda P, Delgado T, Portela A, Melero JA, Garcia-Barreno B (1991). Premature stop codons in the G glycoprotein of human respiratory syncytial viruses resistant to neutralization by monoclonal antibodies.. J Virol.

[44] Lazar I, Canaan A, Weibel C, Kahn JS (2006). Novel mutations in the respiratory syncytial virus G gene identified in viral isolates from a girl with severe combined immune deficiency treated with intravenous immune globulin.. J Clin Virol.

[45] Deng J ZR, Qian Y, Zhao L, Wang F (2006). Sequence analysis of G glycoprotein of human respiratory syncytial virus subtype B strains isolated from children with acute respiratory infections in Beijing, China in years 2000-2004.. Chin J Microbiol Immunol.

[46] Salter A, Laoi BN, Crowley B (2011). Molecular epidemiology of human respiratory syncytial virus subgroups A and B identified in adults with hematological malignancy attending an Irish hospital between 2004 and 2009.. J Med Virol.

[47] Parveen S, Sullender WM, Fowler K, Lefkowitz EJ, Kapoor SK (2006). Genetic variability in the G protein gene of group A and B respiratory syncytial viruses from India.. J Clin Microbiol.

[48] Venter M, Collinson M, Schoub BD (2002). Molecular epidemiological analysis of community circulating respiratory syncytial virus in rural South Africa: Comparison of viruses and genotypes responsible for different disease manifestations.. J Med Virol.

[49] Reiche J, Schweiger B (2009). Genetic variability of group A human respiratory syncytial virus strains circulating in Germany from 1998 to 2007.. J Clin Microbiol.

[50] Johnson PR, Olmsted RA, Prince GA, Murphy BR, Alling DW (1987). Antigenic relatedness between glycoproteins of human respiratory syncytial virus subgroups A and B: evaluation of the contributions of F and G glycoproteins to immunity.. J Virol.

[51] Trento A, Casas I, Calderon A, Garcia-Garcia ML, Calvo C (2010). Ten years of global evolution of the human respiratory syncytial virus BA genotype with a 60-nucleotide duplication in the G protein gene.. J Virol.

[52] Galiano MC, Palomo C, Videla CM, Arbiza J, Melero JA (2005). Genetic and antigenic variability of human respiratory syncytial virus (groups a and b) isolated over seven consecutive seasons in Argentina (1995 to 2001).. J Clin Microbiol.

[53] Kuo L, Fearns R, Collins PL (1996). The structurally diverse intergenic regions of respiratory syncytial virus do not modulate sequential transcription by a dicistronic minigenome.. J Virol.

[54] Kolakofsky D, Pelet T, Garcin D, Hausmann S, Curran J (1998). Paramyxovirus RNA synthesis and the requirement for hexamer genome length: the rule of six revisited.. J Virol.

[55] Kawano M, Okamoto K, Bando H, Kondo K, Tsurudome M (1991). Characterizations of the human parainfluenza type 2 virus gene encoding the L protein and the intergenic sequences.. Nucleic Acids Res.

[56] Moudy RM, Harmon SB, Sullender WM, Wertz GW (2003). Variations in transcription termination signals of human respiratory syncytial virus clinical isolates affect gene expression.. Virology.

[57] Henrickson KJ, Hoover S, Kehl KS, Hua W (2004). National disease burden of respiratory viruses detected in children by polymerase chain reaction.. Pediatr Infect Dis J.

