# Supplementary figures and images for: Whole Genome Sequencing and Evolutionary Analysis of Human Respiratory Syncytial Virus A and B from Milwaukee, WI 1998-2010
Source: PLoS One. 2011 Oct 6;6(10):e25468. doi: 10.1371/journal.pone.0025468 (PMC3188560; doi:10.1371/journal.pone.0025468)

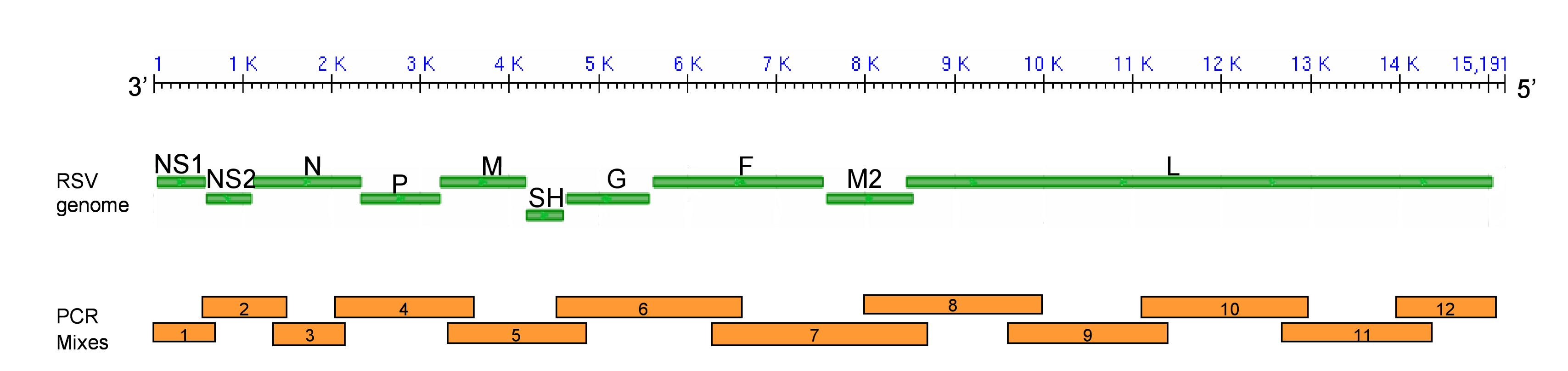

Supplement: Figure S1 — RNA negative-sense genome and amplification strategy of the whole genome of RSV. At the top the kilobase scale is aligned relative to the RSV genome (approximately to scale). Genes are represented as green rectangles named according to the encoded protein. At the bottom the 12 PCR products resulted from the cDNA amplification with 12 overlapping primer sets spanning the entire RSV genome. (TIF) [file pone.0025468.s001.tif]
