# Supplementary material for: Whole Genome Sequencing and Evolutionary Analysis of Human Respiratory Syncytial Virus A and B from Milwaukee, WI 1998-2010
Source: PLoS One. 2011 Oct 6;6(10):e25468. doi: 10.1371/journal.pone.0025468 (PMC3188560; doi:10.1371/journal.pone.0025468)
Supplement: Table S1 — Primers used for amplification and sequencing. (DOC) [file pone.0025468.s002.doc]

**Table S1. Primers used for amplification and sequencing.**

| **Amplification Primers^a^** | | | | | | |
| --- | --- | --- | --- | --- | --- | --- |
| **Segment** | **Forward Primer (5’🡪3’)** | | **Reverse Primer (5’🡪3’)** | | **Locations^b^** | |
| 1 | AAAAAATGCGTACTACAAACTTGC | | GCCATATTTTGTRTTRTATTCAG | | 6-894 | |
| 2 | TTRTTTGACAATGAYGAAGTAGCATTGT | | GCAAARATYCCTTCAACTCTA | | 144-1870 | |
| 2 | CAAAGAYTGATGATCACAGAC | | GCAAARATYCCTTCAACTCTA | | 658-1870 | |
| 3 | TTGAGATAGAATCTAGAAARTCCTAC | | GCATCTTCTCCATGRAATTC | | 1519-2384 | |
| 4 | GCYGGYCTAGGCATAATG | | GCTCCTTTRTTGTCAGTMACTGTG | | 2124-3866 | |
| 4 | GCYGGYCTAGGCATAATG | | TGCTCCTTTRTTGTCAGT | | 2124-3867 | |
| 5 | AYCCTGCATCACTYACAAT | | GTTGTTRGTGTRACTTTGT | | 3314-4875 | |
| 6 (A)^c^ | CAACGCACCGCCAAGACACTAGAA | | GTTACACCTGCATTGACACTAAATTC | | 4676-6361 | |
| 6 (B) | CAACGCACTGCCAGGACTCTAGAA | | GTTACACCTGCATTGACACTAAATTC | | 4676-6361 | |
| 6 | CTGGGAYACTCTYAATCAT | | TTTCCARCARGGTGTATCTAT | | 4705-6575 | |
| 6 | CTGGGAYACTCTYAATCAT | | ATGTCTTTATGATTCCACGAT | | 4705-6933 | |
| 6-7 (A) | CTATCCACAAACAAGGCTGTAGTCAG | | GGGTTATTGATGGTTATGCTCTTG | | 6144-8101 | |
| 6-7 (B) | CTGTCTACAAACAAAGCTGTAGTCAG | | GGGTTGCTTATGGTTATGCTTTTG | | 6144-8101 | |
| 7 | ATGCCTATAACAAATGACCAGAAAA | | ATCACTAATTTCTATGGCTCTTC | | 6420-8829 | |
| 7 | GTCTATAATAAAGGAAGAAGTCCTTGC | | ATAAACRCTGGTGGTTATCC | | 6497-8278 | |
| 7 | ATCGTGGRATYATAAAGACAT | | ATCACTAATTTCTATGGCTCTTC | | 6913-8829 | |
| 7 | GTGTAGTTGGAGTGCTAGAGAGTT | | AACCATGATGGAGGATGTTGCAT | | 7798-9077 | |
| 8 | CCYAAGATAAGAGTGTACAATACTG | | CTTAAGGTAGGCCATCTGTTG | | 7941-9917 | |
| 9 | CTTTATTRGACAAGACAGTGTCTG | | CTCCTGTGTTAAGCTRCCTATAG | | 9611-11208 | |
| 9 | ATTRATTAAGCTTGCAGGTGA | | ATCATCAAGTATHGTRTTTATCCA | | 9684-11166 | |
| 9 | ATTRATTAAGCTTGCAGGTGA | | TTATTGTTACATAACGCATGATTT | | 9684-11315 | |
| 10 | CTAATATCTCTCAAAGGGAAATTC | | CCCTCTCCCCAATCTTTTTC | | 10849-13028 | |
| 11 | TAACAGAAAAGTATGGTGATGAAG | | GAACAGTACTTGCATTTTCTTAC | | 12581-14354 | |
| 12 | CTTTACTGCATGCTTCCTT | | AAGTGTCAAAAACTAATATCTCGT | | 13891-15180 | |
| **Sequencing Primers** | | | | | | |
| **Forward Primers (5’🡪3’)** | | **Position** | | GTACTAATTTAGCTGGACATTGG | | 12944 |
| YRAAAAAATGGGGCAAATAAGA | | 36 | | CTTGGGTTGTTAACATAGATTATC | | 13337 |
| TTRTTTGACAATGAYGAAGTAGC | | 144 | | TATAGATCATTCAGGYAATACAGC | | 13803 |
| ATGATCACRGACATGAGACC | | 667 | | CTTTACYGCATGCTTCCTT | | 13891 |
| CTGAATAYAAYACAAAATATGGC | | 872 | | CYARTTGTATAGCATTCATAGGTGA | | 14003 |
| ATTGAYACTCCYAATTATGATGTGC | | 1236 | | GTAAGAAARTGCAAGTACTGTTC | | 14332 |
| CTTACAGCYGTRATTAGGAG | | 1674 | | GCTCAAGATGAYATTGATTTC | | 14392 |
| GAATTYCATGGAGAAGATGC | | 2365 | | ACCYTTYCTTTGTTACCCTAT | | 14631 |
| GAAACYATAGARACATTTGATAAC | | 2656 | | **Reverse Primers (5’🡪3’)** | | **Position** |
| TAGARGCTATGGCAAGACT | | 2921 | | TTCCCATATGTATCCTCCRTTTTGTA | | 371 |
| CAARGGACCTTCACTARGAGTC | | 3426 | | TTAGGCCATTGGGTTGAGAGCAGT | | 411 |
| TATAACAACCACTGAATTCAAAAATG | | 3768 | | GTTATCAAATGTYTCTATRGTTTC | | 2679 |
| ACTGACAAYAAAGGAGCA | | 3850 | | CTTTTGCCATYTTTTCRCTTTC | | 2971 |
| ATCTTGGRGCKTACCTAG | | 3905 | | GTTGTTGTGTTTTTGATCTTGACT | | 5070 |
| TGGCCYTACYTTACACTAATACA | | 4315 | | GTGGTTTTGTGCTTGGCTTG | | 5108 |
| GCAAATGCAAACATGTCCAAAA | | 4646 | | ACAAAGTTGAACACTTCAAA | | 5169 |
| CAACGCACCGCTAAGACACTAGAA | | 4676 | | GTTGGATTGTTGCTGCATAT | | 5199 (A) |
| TTTGGCAATGATAATCTCAAC | | 4792 | | AGTTGATTGTTGCCACATAT | | 5199 (B) |
| ACAAAGTYACACYAACAAC | | 4857 | | GGTTTGTTTTGTGGTTTGTTTTGG | | 5396 |
| CTGGGGCAAATAACRATGGA | | 5616 | | TCCATTGTTATTTGCCCCAG | | 5635 |
| CTGTCTACAAACAAAGCTGTAGTCAG | | 6144 | | GTTATRACACTRGTATACCAACC | | 5803 |
| ATGCCTATAACAAATGAYCAGAAAA | | 6420 | | GTTACACCTGCATTGACACTGAATTC | | 6361 |
| CAAGRACTGAYAGAGGATGGT | | 6634 | | ATGTCTTTATRATYCCACGAT | | 6933 |
| TGATGCATCAATATCTCAAGTC | | 7094 | | TTGCTTAGTGTRACTGGTGTG | | 7312 |
| CTGAATAAAAATAGCACCTAATCAT | | 7352 | | TGACAGTATTGTACACTCTTA | | 7968 |
| TAGATAACCCATCTATCATTGGAT | | 7405 | | TTCTTCAATACGTCTGCTGG | | 8050 |
| GAGGTCATTGCTTGAATGGTA | | 7609 | | GTTATGCTTTTGTGGATATCCAATGT | | 8089 |
| TCWGAAATAAGTGGAGCTGC | | 7746 | | GGGTTGTTGATGGTTATGCTCTTG | | 8101 |
| GTGTAGTTGGAGTGCTAGAGAGTT | | 7798 | | TTGTATGTCATAAGTAATGACTG | | 8750 |
| GGACAAAATGGATCCCATTATT | | 8460 | | ATCACTRATTTCTATRGCTCTTC | | 8829 |
| AAGGTGTTATYTCTTTYTCAGA | | 8525 | | AATTTCTTCAAGAGTGTTGTTTTGAT | | 9044 |
| GTGTTCAATGCARCATCC | | 9048 | | AACCATGATGGAGGATGTTGCAT | | 9077 |
| CCATCATGGTTAATACAYTGGTT | | 9067 | | RCAAACATTTAATCTRCTAAGGC | | 9315 |
| AGARGTAGAGGGATTTATTATGT | | 9465 | | CTTAARGTRGGCCATCTGTTG | | 9917 |
| TCACAYATACAAAAYTATATAGAACATG | | 10144 | | GATTGTACRCCATGCAGTTC | | 10649 |
| AGTYTKACAAGATATGGTGATCT | | 10438 | | TTATTGTKACATAAYGCATGATTT | | 11315 |
| CTAATATCYCTCAAAGGGAAATTC | | 10849 | | RGGATCACCACCACCAAA | | 11436 |
| AAGTGAGTYTAGAATCTATAGGTAG | | 11171 | | CGATATAACAARTTRGGATCAC | | 11450 |
| TTATTATGCAGTTTAATATTTAG | | 11230 | | CTAGTAATTTTAGCTTGCCT | | 11687 |
| GTGATCCYAAYTTGTTATATCG | | 11429 | | GAGAATATTTTGTTTGGAGCT | | 11747 |
| TTYTCYAAAAGTGCACAACATT | | 11743 | | TGCAGGGAATTCACATGG | | 12519 |
| ACTTACCCTCATGGATTAAGAG | | 11815 | | CTTGGGTTGTTAACATAGATTATC | | 13360 |
| GCYACTGARATGATGAGG | | 11959 | | GAACAGTACTTGCAYTTTCTTAC | | 14354 |
| GTWAGRGAAAGATCTTGGTC | | 12082 | | CTTCATTYCGTCCYGCTATAG | | 14743 |
| TAACAGAAAAGTATGGYGATGAAG | | 12581 | |  | |  |

^a^ Amplification primers were also used for sequencing.

^b^ Locations and positions are for the reference sequence NC_001803. For the sequencing primers the position listed is the 5’ end of the primer.

^c^ Some primer pairs or individual primers were designed specifically for RSV A or B.
